# Comparison of Optic Disc Ovality Index and Rotation Angle Measurements in Myopic Eyes Using Photography and OCT Based Techniques

**DOI:** 10.3389/fmed.2022.872658

**Published:** 2022-06-24

**Authors:** Jasmin Rezapour, Andrew Q. Tran, Christopher Bowd, Nevin W. El-Nimri, Akram Belghith, Mark Christopher, Nicole Brye, James A. Proudfoot, Jade Dohleman, Massimo A. Fazio, Jost B. Jonas, Robert N. Weinreb, Linda M. Zangwill

**Affiliations:** ^1^Viterbi Family Department of Ophthalmology, Hamilton Glaucoma Center, Shiley Eye Institute, University of California, San Diego, San Diego, CA, United States; ^2^Department of Ophthalmology, University Medical Center of the Johannes Gutenberg University Mainz, Mainz, Germany; ^3^Department of Ophthalmology, School of Medicine, The University of Alabama at Birmingham, Birmingham, AL, United States; ^4^Department of Biomedical Engineering, School of Engineering, The University of Alabama at Birmingham, Birmingham, AL, United States; ^5^Department of Ophthalmology, Medical Faculty Mannheim, Heidelberg University, Mannheim, Germany; ^6^Institute of Molecular and Clinical Ophthalmology, Basel, Switzerland

**Keywords:** high myopia, glaucoma, OCT, Bruch's membrane opening, axial myopia, optic nerve head morphology, clinical disc margin, OCT landmarks

## Abstract

**Purpose:**

To compare optic nerve head (ONH) ovality index and rotation angle measurements based on semi-automated delineation of the clinical ONH margin derived from photographs and automated BMO configuration derived from optical coherence tomography (OCT) images in healthy and glaucomatous eyes with high-, mild- and no axial myopia.

**Methods:**

One hundred seventy-five healthy and glaucomatous eyes of 146 study participants enrolled in the Diagnostic Innovations in Glaucoma Study (DIGS) with optic disc photographs and Spectralis OCT ONH scans acquired on the same day were stratified by level of axial myopia (non-myopic [*n* = 56, axial length (AL) <24 mm], mild-myopic [*n* = 58, AL 24–26 mm] and high-myopic [*n* = 32, AL >26 mm]. The clinical disc margin of each photograph was manually annotated, and semi-automated measurements were recorded of the ovality index and rotation angle based on a best-fit ellipse generated using ImageJ software. These semi-automated photograph-based measurements were compared to ovality index and rotation angle generated from custom automated BMO-based analysis using segmented OCT ONH volumes. *R*^2^ values from linear mixed effects models were used to describe the associations between semi-automated, photograph-based and automated OCT-based measurements.

**Results:**

Average (95% CI) axial length was 23.3 (23.0, 23.3) mm, 24.8 (24.7, 25.0) mm and 26.8 (26.6, 27.0) mm in non-myopic, mild-myopic and high-myopic eyes, respectively (ANOVA, *p* ≤ 0.001 for all). The *R*^2^ association (95% CI) between semi-automated photograph-based and automated OCT-based assessment of ONH OI for all eyes was [0.26 (0.16, 0.36); *p* < 0.001]. This association was weakest in non-myopic eyes [0.09 (0.01, 0.26); *p* = 0.02], followed by mild-myopic eyes [0.13 (0.02, 0.29); *p* = 0.004] and strongest in high-myopic eyes [0.40 (0.19, 0.60); *p* < 0.001]. No significant associations were found between photography- and OCT-based assessment of rotation angle with *R*^2^ values ranging from 0.00 (0.00, 0.08) in non-myopic eyes to 0.03 (0.00, 0.21) in high-myopic eyes (all associations *p* ≥ 0.33).

**Conclusions:**

Agreement between photograph-based and automated OCT-based ONH morphology measurements is limited, suggesting that these methods cannot be used interchangeably for characterizing myopic changes in the ONH.

## Introduction

Axial myopia typically results from progressive eye enlargement, reflected in a larger axial length (AL). The prevalence of myopia has increased in the United States (1) and worldwide (2), with some populations reporting prevalence as high as 96% (3). It is now projected that ~50% of the world's population will be myopic by 2050, with 10% being highly myopic (4). There is strong evidence linking myopia with glaucoma (5). For instance, the Blue Mountains Eye Study reported that compared to eyes with no myopia, the odds of having glaucoma in mild myopic eyes was 2.3, and this increased to 3.3 for moderate to high myopic eyes (6). This association presents significant public health concerns in the context of swiftly rising myopia prevalence. Moreover, accurate detection of glaucoma in myopic patients is challenging. Myopic eyes often develop structural changes at the optic nerve head (ONH) independent of glaucoma (7, 8), and these changes are often difficult to distinguish from those observed in glaucoma.

Many studies have reported the prevalence of ONH ovality, tilt and torsion and their associations with myopia, particularly with high myopia (9–20). However, these studies often used manual methods to measure ovality, tilt and torsion parameters based on measurements of two- dimensional fundus photographs or two-dimensional fundus photographs projected on spectral-domain optical coherence tomography (OCT) scans. Although manual or semi-automated measurement of ovality, tilt and torsion from photographs are the most common methods reported in the literature, they are not based on anatomical landmarks such as Bruch's membrane opening (BMO). Furthermore, two-dimensional viewing of the ONH at an oblique angle may lead to inaccurate observations with regard to the shape of the disc, especially its horizontal diameter (19). We previously reported an automated custom-software based method to measure ONH ovality index, tilt and rotation (torsion) angles based on the BMO assessed on OCT scans (21). The purpose of the current study is to compare semi-automated measurements of optic disc ovality index (often referred to as tilt) and rotation angle based on the manually demarcated clinical disc margin identified on optic disc stereophotographs with automated measurements based on the BMO identified from ONH-OCT scans in eyes with no axial myopia, mild axial myopia and high axial myopia, with and without glaucoma.

## Materials and Methods

This cross-sectional comparison of semi-automated photograph-based vs. automated OCT-based assessment involved healthy individuals and glaucoma patients with varying levels of axial myopia, from the Diagnostic Innovations in Glaucoma Study (DIGS). The DIGS is an ongoing prospective, longitudinal study conducted at the Hamilton Glaucoma Center, University of California, San Diego, designed to evaluate anatomical structures in glaucoma. Details of the DIGS protocol have been described elsewhere (22). All methods adhered to the tenets of the Declaration of Helsinki and the Health Insurance Portability and Accountability Act and were approved by the Institutional Review Board of the University of California, San Diego.

### Participants

Study participants were a subset of glaucoma patients and healthy individuals with varying degrees of axial myopia enrolled in DIGS (clinical trials.gov identifier NCT00221897). All participants were ≥18 years old, had open anterior chamber angles, and had undergone a full ophthalmologic examination including refractometry, best-corrected visual acuity assessment, standard automated perimetry [Humphrey Field Analyzer; 24–2 Swedish interactive thresholding algorithm (SITA) standard; Carl-Zeiss Meditec], Goldmann applanation tonometry, gonioscopy, dilated fundus examination, central corneal thickness (CCT) measurement by ultrasound pachymetry (DGH Technology, Inc., Exton, PA), coherence interferometry measurement of the axial length (IOLMaster, Carl Zeiss Meditec, Dublin, CA), and simultaneous stereophotography and OCT imaging of the optic disc and macula.

Patients with primary open angle glaucoma (POAG) were clinically defined by the DIGS conventional standard of visual field loss or photograph-based optic disc damage (22)^30^.

Standard automated perimetry glaucomatous visual field damage was defined as two repeatable and reliable visual field tests (rate of fixation losses and false negatives and false positives responses of <33%) with a glaucoma hemifield test (GHT) outside normal limits and/or a pattern standard deviation (PSD) with a *p*-value of <0.05 with a similar defect on consecutive abnormal tests (22). Healthy subjects were required to have intraocular pressure ≤ 21 mmHg and no structural abnormalities or functional loss. The Visual Field Assessment Center (VisFACT) Reading Center completed the quality control of all visual fields according to standard protocols and excluded unreliable visual field tests (22).

Stereophotograph-based optic disc damage was defined as focal or diffuse narrowing of the neuroretinal rim, and/or detection of RNFL defects characteristic of glaucoma based on masked assessment by two trained reviewers (JR and CB) after a high myopia optic disc grading training with a senior consultant (JBJ). Optic disc damage was defined by consensus between both graders and in case of disagreement, diagnosis was defined by adjudication by the senior consultant. Intergrader agreement was good with agreement on 131/175 (76.6%) eyes; 41/175 (23.4%) required consensus meetings between the two graders to determine diagnosis. Adjudication by the senior consultant was needed in 3/175 (1.7%) of eyes.

### Axial Myopia Categories

Elongation of the globe in the axial plane can lead to morphological changes of the optic disc and the fundus.^32, 33^ Such elongation and associated changes are not always directly correlated with myopia defined by the refractive error. Furthermore, refractive error is subject to change after patients undergo cataract surgery or refractive procedures. In this study we were interested in morphological changes of the optic disc due to axial elongation and for these reasons myopia was defined by axial length and not by refractive error. Based on population-based studies (23, 24), axial myopia was stratified into three groups: no axial myopia (AL <24.0 mm), mild axial myopia (AL = 24.0–26.0 mm) and high axial myopia (AL >26.0 mm). For each eye, ovality and rotation angle were measured based on: (1) stereophotographs using manual annotation of the clinical disc margin and (2) OCT images of the ONH using automated analysis of BMO.

### Image Acquisition

Simultaneous stereophotographs centered on the optic disc were obtained for all participants. Poor quality images due to low lighting, haziness or otherwise obscured features of the optic disc were excluded.

ONH radical circle (ONHRC) scans from the Spectralis Glaucoma Module Premier Edition (version 6.10; Heidelberg Engineering Inc., Heidelberg, Germany) were acquired on all participants. The ONHRC scan consists of 24 high-resolution ONH radial scans and 3 RNFL circle scans. Images were electronically transferred for quality assessment to the UC San Diego Imaging Data Evaluation and Analysis (IDEA) Reading Center. Low quality images, with quality score <15, or poor centering (based on the distance between the BMO center of the ONHRC scans and the center of the image) were excluded.

### Semi-automated Photograph-Based Measurement of the Clinical Optic Disc Margin and Optic Disc Ovality Index and Rotation Angle

**Figure 1:**
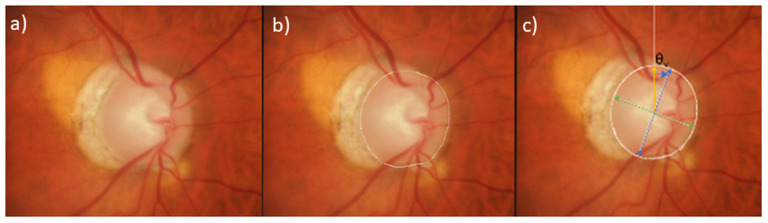
Delineation of semi-automated measurement of ovality index and rotation angle from photographs. **(a)** Stereophotograph of a glaucomatous mild myopic eye, **(b)** Clinical disc margin manually delineated in white, **(c)** Best-fitted ellipse overlaid in white. Ovality index is calculated as the minor axis (green) divided by the major axis (blue). Rotation angle (θ_v_ blue arrow) is defined as the angle between the vertical axis (yellow) and major axis (blue).

Digital stereophotographs were reviewed through a stereo viewer and annotated manually in ImageJ (ver. 1.53a, NIH) on a touch-screen tablet. The photographs of left eyes were transformed into right eye format for all analyses. The clinical disc margin, defined as the inner margin of the scleral ring was manually demarcated by one study author (AT) and its location was confirmed by all 3 senior reviewers (JR, CB, NEN). A best-fit ellipse was generated by ImageJ. From the best-fit ellipse, the length of the major (longest) axis, minor (shortest) axis, and ultimately the angle between the major axis and the vertical axis were automatically generated by ImageJ and exported to.csv files to calculate ovality index and rotation angle and direction (Figure 1). Annotations were made on the image and the best-fit ellipse from which the measurements were derived and saved for later review to confirm proper selection of the disc margin as well as appropriate calculation of rotation angle and direction.

**Figure 2:**
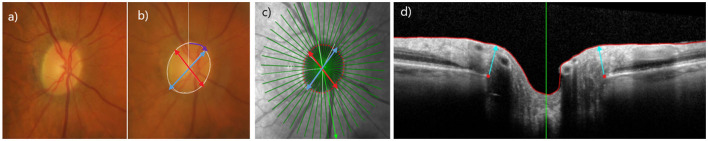
Ovality and Rotation angle measurements on photographs and OCT. **(a)** Stereophotograph of a healthy eye, **(b)** clinical disc margin annotated in white, labeled with major axis (blue), minor axis (red), rotation angle (purple); Ovality index (minor axis/major axis), **(c)** en-face OCT optic nerve head image of Bruch's Membrane Opening (BMO) (red); Ovality index (minor axis (red)/major axis (blue). Please note as described previously, the calculations use the 3D OCT scan. (21) **(d)** b-scan of optic nerve head with BMO points in red.

As described in our previous study (21), optic disc ovality index can be defined on a 2- or 3-dimensional level, whereas optic disc tilt is calculated on a 3-dimensional level. As photograph-based measurements can only be performed on a 2-dimensional level, we assessed the optic disc ovality index and not the optic disc tilt angle in this report. Ovality index was calculated by dividing the minor axis by the major axis, with a value closer to 0 indicating a more oval optic disc. For this report, we used the term “rotation” rather than the more commonly used term “torsion,” as “torsion” implies shearing of the tissue that can only be detected at a histological level (25). The optic disc rotation angle was defined as the smaller angle formed by either the major axis or minor axis and the vertical axis extending from the center of the best-fit ellipse (Figure 2). We adjusted for whether a right eye or left eye was analyzed, and conserved the magnitude of the rotation angle, regardless of the direction.

### Automated OCT-Based BMO Ovality Index and Rotation Angle Measurements

Ovality index and rotation angle measurements based on BMO points were calculated for the segmented Spectralis ONHRC scans using the San Diego Automated Layer Segmentation Algorithm (SALSA) Image Processing Pipeline. Details of SALSA and its validation have been described previously (21, 26, 27). In brief SALSA is a python-based automated algorithm that segments retinal layers in OCT scans. Spectralis OCT raw image (vol) files were exported to the SALSA-Image Processing Pipeline. SALSA identified the Bruch's membrane (BM) and the 2 BMO points in each scan, produced a fitted ellipse and documented the results as a.csv file. The accuracy of the BMO locations was reviewed by one of the authors (JR) on a randomly chosen subset of 30 non-, mild- and high axial myopic eyes and showed good performance. Ovality index was defined as the minor axis (shortest axis) divided by the major axis (longest axis) of the fitted BMO ellipse.

The OCT-based rotation angle is defined as the angle between the major axis and the temporal axis (0°, horizontal axes of the *enface* OCT image). In another words, we characterize rotation as the angle of rotation around the sagittal axis (z axis). Angles are reported after transforming the vectors used in their computations into physical space by scaling according to the scale x and scale z constants in the image volume metadata as described previously (26, 27). All measurements were calculated with respect to individual anatomies by factoring in the Fovea-BMO center (FoBMOc) angle captured by the Spectralis OCT.

Details of how the automated BMO ovality index and rotation angle measurements were completed have been described previously (21).

### Statistical Analyses

Statistical analysis was conducted to compare ONH ovality index and rotation angle values determined by semi-automated assessment of photographs and automated assessment of OCT images. Data is presented as mean (95% confidence interval) or count (percentage) for continuous and categorical variables, respectively. The statistical significance of comparisons between patient-level characteristics across myopia groups was determined by analysis of variance (ANOVA) for continuous variables and Fisher's exact test for categorical variables. For eye-level characteristics, mean and confidence interval estimates were derived from linear mixed effect models, with a random intercept to account for within-subject correlation before and after adjusting for age and visual field mean deviation. Linear mixed effect models were used to compare semi-automated-derived ovality index and rotation angle values (separately) from photographs with those automatically derived from OCT images, stratified by axial myopia. R-squared values for reported for correlation between semi-automated photograph-based and automated OCT-based ovality index and rotation angle. Bland-Altman plots were used to characterize the agreement between photograph- and OCT-based measurements of ovality index and rotation angle. We considered *p*-values <0.05 to indicate statistical significance throughout. All statistical analyses were performed using the R (version 3.5.2).

## Results

### Demographics and Clinical Characteristics

One hundred and seventy-five eyes from 146 patients were included in this study, with 64 eyes (56 patients) in the no axial myopia group, 70 eyes (58 patients) in the mild axial myopia group and 41 eyes (32 patients) in the high myopia group (Table 1). The no myopia and mild axial groups have a larger proportion of healthy participants than the high axial myopia group (42.2, 41.4, and 26.8%, respectively). The mean (95% CI) age of the participants in the no axial myopia group was older [70.7 (67.3, 74.1)] years than those in the mild [65.2 (61.1, 69.4)] years, followed by the high [63.3 (58.0, 68.7)] years axial myopia groups (*p* < 0.040). Mean (95% CI) spherical equivalent was significantly lower in high axial myopic eyes [−4.01 (−4.78, −3.23)] D compared to that of eyes with mild [−1.51 (−2.09, −0.93)] D or no axial myopia [0.09 (−0.50, 0.69)] D (visual field MD/age adjusted; *p* < 0.001). There was no significant difference in the visual field MD (*p* = 0.27), pattern standard deviation (PSD) (*p* = 0.37), BMO area (*p* = 0.15), or CCT (*p* = 0.12) among myopia groups.

**Table 1:** Patient characteristics by myopia status.

	**No myopia** **(*n* = 56)**	**Mild myopia** **(*n* = 58)**	**High myopia** **(*n* = 32)**	**Overall** **(*n* = 146)**	***P*-value**	**MD/Age Adj. *p*-value**
**Patient specific data**						
Age (years)	70.7 (67.3, 74.1)	65.2 (61.1, 69.4)	63.3 (58.0, 68.7)	66.9 (64.5, 69.3)	0.04	
**Sex**						
Female	39 (69.6%)	31 (53.4%)	12 (37.5%)	82 (56.2%)	0.01	
Male	17 (30.4%)	27 (46.6%)	20 (62.5%)	64 (43.8%)		
**Race**						
Asian	5 (8.9%)	6 (10.3%)	11 (34.4%)	22 (15.1%)	0.02	
Black or African American	16 (28.6%)	20 (34.5%)	4 (12.5%)	40 (27.4%)		
White	32 (57.1%)	31 (53.4%)	16 (50.0%)	79 (54.1%)		
Unknown or not reported	3 (5.4%)	1 (1.7%)	1 (3.1%)	5 (3.4%)		
**Eye specific data**						
BMO area (mm^2^)	*n* = 64 2.12 (1.97, 2.27)	*n* = 68 2.20 (2.05, 2.35)	*n* = 38 2.37 (2.17, 2.58)	*n* = 170 2.20 (2.11, 2.30)	0.15	0.24
Axial length (mm)	*n* = 64 23.2 (23.0, 23.3)	*n* = 70 24.8 (24.7, 25.0)	*n* = 41 26.8 (26.6, 27.0)	*n* = 175 24.6 (24.4, 24.9)	<0.001	<0.001
Spherical equivalent (Dpt)	*n* = 64 0.09 (–0.50, 0.69)	*n* = 70 –1.51 (–2.09, –0.93)	*n* = 41 –4.01 (–4.78, –3.23)	*n* = 175 –1.44 (–1.88, –1.00)	<0.001	<0.001
Eye classification	*n* = 64	*n* = 70	*n* = 41	*n* = 175	-	-
Healthy	27 (42.2%)	29 (41.4%)	11 (26.8%)	67 (38.3%)		
Glaucoma	37 (58.8%)	41 (58.5%)	30 (73.1%)	108 (61.7%)		
Visual field MD (dB)	*n* = 64 –3.14 (–4.72, –1.55)	*n* = 70 –3.89 (–5.45, –2.34)	*n* = 41 –5.29 (–7.38, –3.21)	*n* = 175 –3.91 (–4.89, –2.93)	0.27	0.08

*Results are presented as mean (95% CI) or counts (percentages)*.

### Ovality Index and Rotation Angle Measurements

Overall, we found that the mean ovality index values derived from photographs were similar to those derived from OCT in each myopia category (Table 2). Specifically, mean (95% CI) photograph-based ovality index was significantly lower (i.e., ONH was more oval) in high axial myopic eyes [0.85 (0.83, 0.88)] compared to eyes with mild [0.90 (0.89–0.92)] or no [0.89 (0.88–0.91)] axial myopia (MD/age adjusted *p* = 0.002). Similarly, mean OCT-based ovality index was significantly lower in high axial myopic eyes [0.84 (0.81, 0.86)] compared to eyes with mild [0.88 (0.85–0.89)] or no [0.88 (0.88–0.90)] myopia (MD/age adjusted *p* = 0.008).

**Table 2:** Ovality Index (closer to 0=more oval, closer to 1=more circular) and Rotation Angle by Myopia Status.

		***No Myopia***	***Mild Myopia***	***High Myopia***	***Overall***	*P*-value*	**Visual field MD and age adjusted *p*-value
Ovality index	Photograph-based	*n* = 64 0.89 (0.88, 0.91)	*n* = 70 0.90 (0.89, 0.92)	*n* = 41 0.85 (0.83, 0.88)	*n* = 175 0.89 (0.88, 0.90)	0.002	0.002
	OCT-based	*n* = 64 0.88 (0.86, 0.89)	*n* = 70 0.88 (0.86, 0.90)	*n* = 41 0.84 (0.81, 0.86)	*n* = 175 0.87 (0.86, 0.88)	0.01	0.008
Rotation angle (degrees)	Photograph-based	*n* = 63 15.2 (11.8, 18.7)	*n* = 69 19.9 (16.6, 23.3)	*n* = 38 20.9 (16.4, 25.4)	*n* =169 18.4 (16.2, 20.5)	0.078	0.108
	OCT-based	*n* = 63 35.9 (31.3, 40.6)	*n* = 68 34.4 (29.9, 39.0)	*n* = 38 33.7 (27.6, 39.9)	*n* = 169 34.8 (32.0, 37.7)	0.83	0.96

*p-value based on linear mixed models adjusting for both eyes in the model. **p-value based on linear mixed models adjusting for age, visual field mean deviation (MD) and both eyes in the model. Results are presented as mean (95% confidence interval).

In contrast, the mean values of OCT-based rotation angles were much larger than photograph-based measures (34.8 and 18.4 degrees, respectively). Moreover, photograph-based rotation angles tended to be larger in high axial myopic eyes (20.9 degrees) compared to eyes with mild (19.9 degrees) and no myopia (15.2 degrees) (visual field MD and age-adjusted *p* = 0.108) but there was no difference in the OCT-based rotation angles across the three categories of myopia (visual field MD and age-adjusted *p* = 0.96.

### Agreement Between Photograph-Based and OCT-Based Measurements of Optic Disc Ovality Index and Rotation Angle

**Figure 3:**
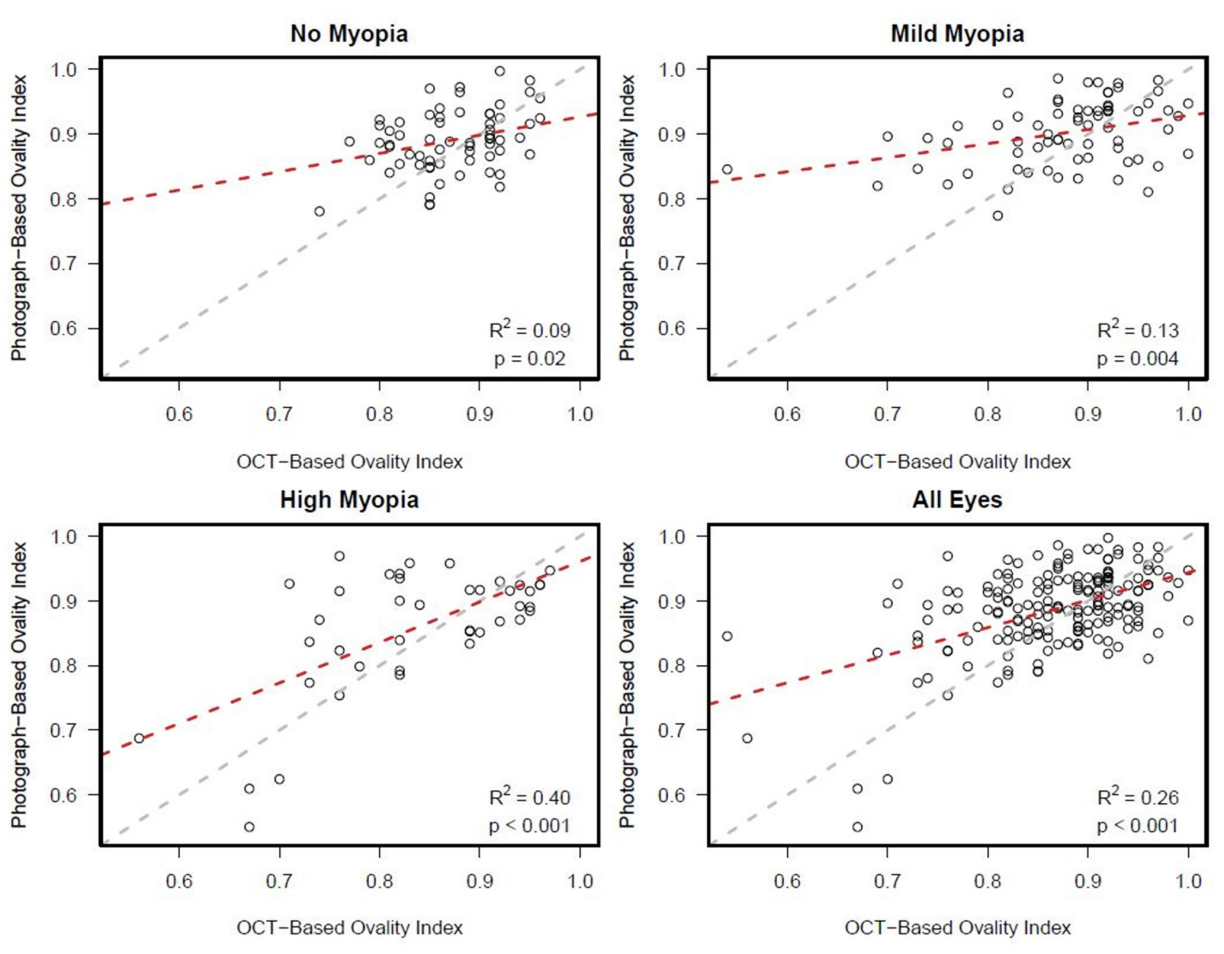
Ovality index: Scatterplots showing stronger associations between semi-automated clinical disc margin photograph-based ovality index and automated, BMO OCT-based ovality index in eyes with axial high myopia compared to eyes with mild or no axial myopia.

**Figure 4:**
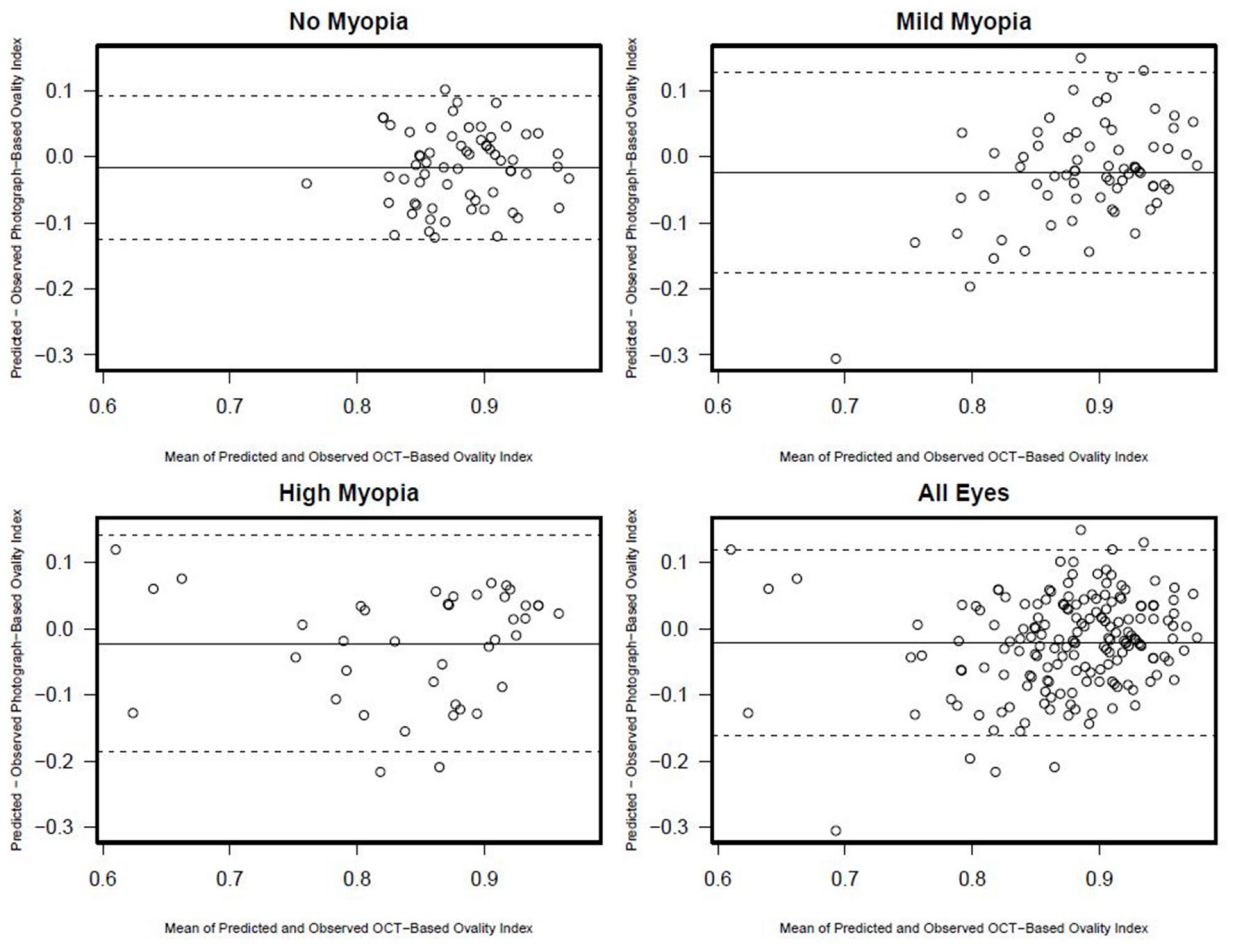
Ovality index Bland-Altman plots documenting the agreement between photograph and OCT-based ovality index by myopia status.

There was a statistically significant association [*R*^2^ (95% CI)] between semi-automated photograph-based and automated OCT-based assessment of ONH ovality index for all eyes [0.26 (0.16; 0.36); *p* < 0.001]. This association was weakest in non-myopic eyes [0.09 (0.01, 0.26); *p* = 0.02], followed by mild-axial myopic eyes [0.13 (0.02, 0.29); *p* = 0.004] and strongest in high-axial myopic eyes [0.40 (0.19, 0.60); *p* < 0.001] (Figure 3). In addition, Bland-Altman plots suggest that the mean difference in the photograph- and OCT-based ovality index values was close to zero and did not vary by the degree of ovality or myopia status (Figure 4).

**Figure 5:**
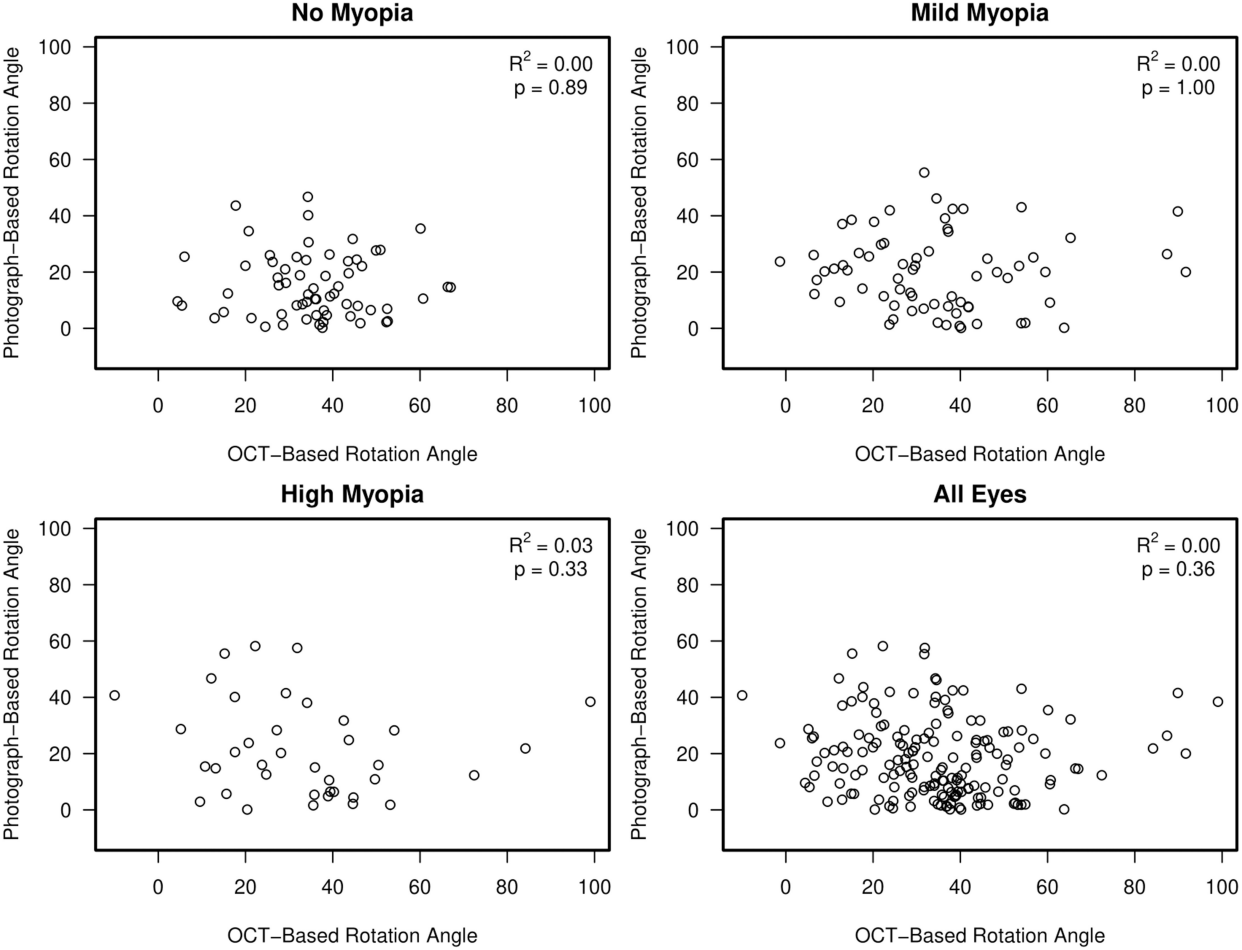
Scatterplots showing no association between semi-automated, clinical disc margin photograph-based rotation angle and automated, BMO OCT-based rotation angle.

**Figure 6:**
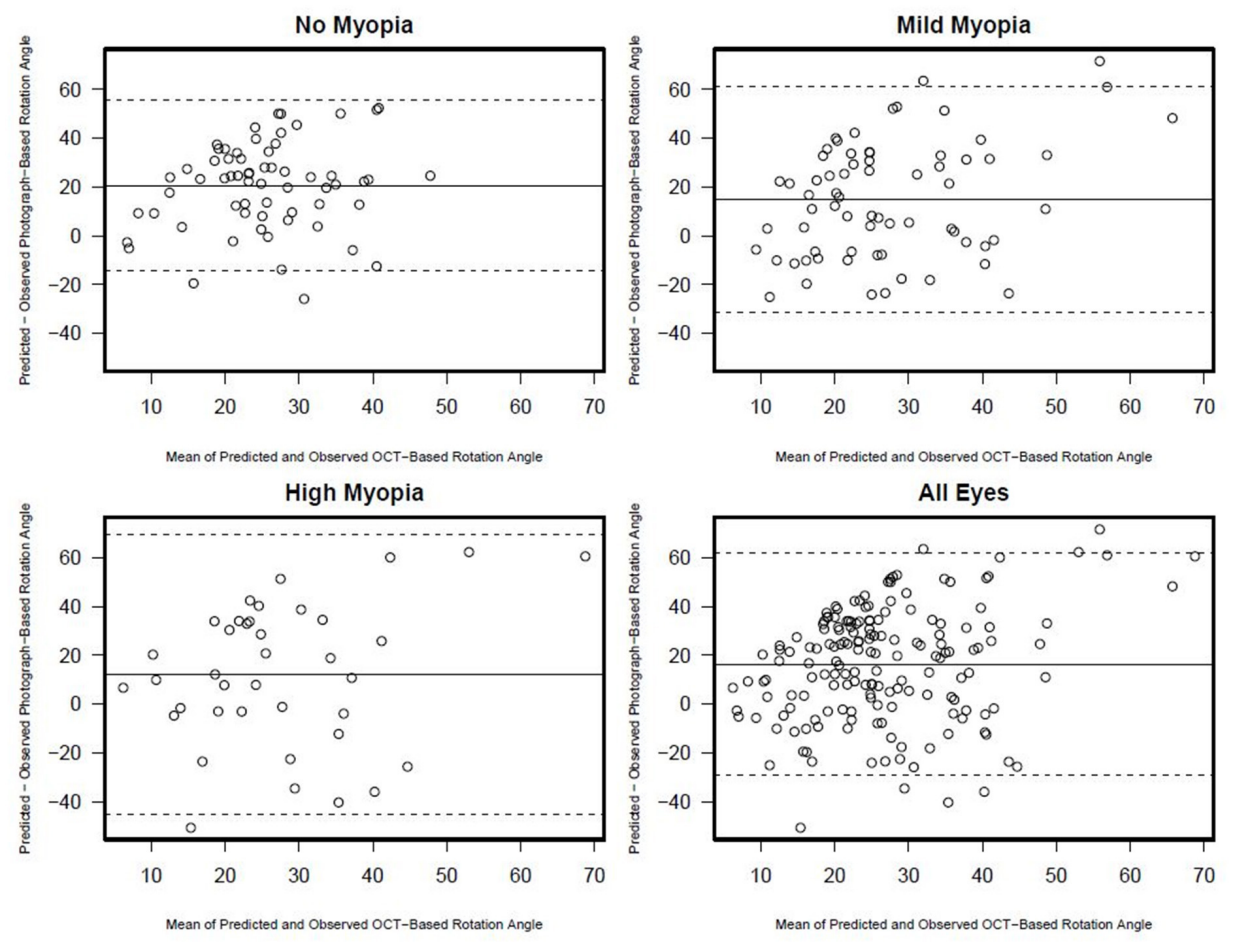
Rotation angle Bland-Altman plots documenting the agreement between photograph and OCT based rotation angle by myopia status.

There was no association between semi-automated photograph-based and automated OCT-based assessment of rotation angle with *R*^2^ values ranging from 0.00 (0.00, 0.08) in non-myopic eyes to 0.03 (0.0, 0.21) in high-axial myopic eyes (all associations *p* ≥ 0.33) (Figure 5). The Bland-Altman plots suggest that the difference in the photograph- and OCT-based rotation angle was close to 20 degrees and did not vary by rotation angle (Figure 6).

## Discussion

To our knowledge, this is the first study to compared semi-automated measurements of ONH ovality index and rotation angle based on the clinical disc margin manually identified on optic disc photographs to automated OCT measurements based on the BMO in healthy and glaucomatous eyes with high-, mild- and no axial myopia. We found a statistically significant, weak to moderate association between manual photograph-based and automated OCT-based assessment of ONH ovality index (*R*^2^ = 0.26), which was strongest in highly myopic eyes (*R*^2^ = 0.40), followed by weaker associations in mild-myopic (*R*^2^ = 0.13), and non-myopic eyes (*R*^2^ = 0.09). No association was found between photograph-based and OCT-based assessment of the rotation angle with *R*^2^ values ranging from 0.00 to 0.03 (all *p* > 0.33). The large difference between photographs and OCT-based rotation angle measurements suggests that these parameters cannot be used interchangeably, and the results should not be directly compared.

Axial elongation can cause pathological changes located at the posterior pole, such as myopic maculopathy, Bruch's membrane defects and posterior staphyloma (28). The clinical challenge to diagnose glaucoma in (highly) myopic eyes is that the appearance of the ONH can mimic changes that are pathognomonic for glaucoma. Those changes include optic disc ovality/tilt, rotation and peripapillary atrophy. At the same time, myopic changes can also mistakenly be interpreted as glaucoma (29). We hypothesize that objective characterization of the morphological characteristics of myopic disks can be used to help identify features related more to myopia than glaucoma (and vice versa). A precise characterization of the myopic optic disc is the first step to elucidate parameters that can ultimately help improve clinicians' ability to differentiate between (high) myopia and glaucoma.

We previously reported methods to characterize the myopic optic disc with an OCT based automated three-dimensional approach to characterize the ONH morphology in myopic eyes (21). We used this automated custom software to measure optic disc ovality index and rotation angle in the current study. Most previous studies characterized the myopic optic disc using manual photograph-based methods (9–13, 18, 30–35). The current study suggests that the characterization of the degree of ovality and rotation of the myopic optic disc based on photographs and OCT is not interchangeable.

It has been reported previously that optic disc features that are assessed two-dimensionally based on the clinical disc margin of optic disc photographs and three-dimensionally based on the BMO from OCT scans often do not agree (36). Based on measurements of fundus photographs relative to the clinical disc margin, myopic optic disks have been reported to have a more oval configuration (11, 18, 30–32, 34, 37), a higher degree of disc tilt (12, 13, 18, 30, 33–35) and rotation (11, 12, 18, 31, 34, 35). The current study confirms greater ovality (lower ovality index) in eyes in the high axial myopia group compared to eyes in the no and mild axial myopia groups using both semi-automated photograph- and automated OCT measurements. However, the rotation angle is larger in the high myopia group, but only when measured from the photographs and not when measured from OCT scans. These differences are in large part due to the fact that the features and anatomy used to delineate optic disc margins from clinical examination or fundus photography are often different from features identified in OCT as the BMO, resulting in different rotation angle values. The OCT-based rotation angle is a 3-dimensional BMO-based assessment while the 2-dimensional photographed-based rotation angle is measured relative to the clinical disc margin, which does not always correspond with the BMO, particularly in myopic eyes. Previous studies co-localized optic disc stereophotographs to OCT images of the optic disc and found that the clinically identified disc margin does not correspond to one single anatomic structure in OCT images (38). For example, in some eyes, in specific regions of the optic disc, Bruch's membrane extends beyond the clinically identified disc margin and is therefore not visible on a photograph or a clinical exam (36, 38, 39). For example in myopic eyes, OCT ONH images often show a temporal displacement of the BMO relative to the anterior scleral opening and the clinical disc margin (40). Another possible explanation for the differences in rotation angle is that there is an error in our automated OCT measurement of rotation angle. To investigate this issue, the rotation angle was calculated using an independent automated and histologically validated OCT method (41). Similar results were observed suggesting strongly that the rotation angle is not larger in eyes of the high myopic group compared to eyes in the mild and no myopia group (data not shown, personal communication with Massimo Fazio—June 2020).

A limitation of photograph-based measurements is that especially in tilted disks, which are more common in high myopic eyes (21), the optic disc is seen as more oval because of the oblique observation angle (33). It should be noted that we use the term rotation (25) instead of the more commonly used term torsion to describe the rotation of the BMO around a sagittal axis (instead of vertical axis, as measured by the 3D tilt angle) (42, 43). In contrast, measuring optic disc configuration based on an objective anatomical BMO-based feature improves our ability to obtain repeatable measurements of the ONH. However, we documented recently, objective detection of the BMO in OCT scans can also be challenging, particularly in some eyes with high axial myopia (44).

Given the results of the current study and the consistent evidence that the subjective delineation of the clinical disc margin often does not represent the anatomical BMO-based disk configuration, particularly in myopic eyes (36, 39), objective measurement and characterization of the optic disc using OCT appears to be a more appropriate tool to objectively characterize the morphology of the optic disc. Characterizing the ONH based on OCT anatomical morphology may facilitate the identification of biomarkers that can help clinicians differentiate between high myopes with and without glaucoma.

This study has several limitations. First, manual measurements were assessed by only one of the authors and we therefore did not provide interobserver variability and reproducibility. However, all measurements were re-evaluated by three expert graders. Second, OCT measurements were based only on Spectralis OCT imaging. Our results are therefore not generalizable to other OCT devices. Third, the horizontal axis for semi-automated photograph-based rotation angle calculation was not defined by the Fovea-BMO axis and therefore was not adjusted for an individual's anatomy of the eye. Fourth, there is a significant difference in age and race within the different myopia groups. However, it is unlikely that this may affect the results, as in this study two different methods were compared in the exact same eyes and results reported separately for non-, mild- and high myopic eyes. Finally, as we included both glaucoma and healthy eyes in the analysis it is possible that glaucomatous optic nerve head changes may have influenced the mean ovality index and rotation angle values. To address this issue, we adjusted for visual field MD in the analysis comparing mean ovality index and rotation angles by myopia status for both the photograph- and OCT-based measurement strategy (Table 2). The significant differences in ovality index across myopia status remained after this adjustment, suggesting that including glaucoma patients in the analysis is not driving the results. Moreover, as the main objective of this report is to compare photograph and OCT-based analysis strategies, regardless of diagnosis, including glaucoma patients may increase the generalizability of the results.

A strength of this study is that myopia groups were defined by AL instead of using the spherical equivalent. Axial length better represents the size of the eye and its axial elongation than spherical equivalent which changes after cataract and refractive surgery. In addition, both photograph-based and OCT-based measurements were automated to improve repeatability. Furthermore, the automated OCT-based ovality index and rotation angle measurements were validated with an independent method.

In conclusion, we found weak to moderate associations between manual photograph-based and OCT BMO-based assessment of optic disc ovality and no association between assessment of optic disc rotation angle. Our results indicate that measurements based on these methods cannot be used interchangeably and results should not be directly compared.

## Data Availability Statement

The datasets analyzed during the current study are not publicly available until the ongoing NIH funded studies are complete, but are available in a de-identified format from the corresponding author on request.

## Ethics Statement

The studies involving human participants were reviewed and approved by Institutional Review Board of the University of California, San Diego, clinicaltrials.gov, identifier: NCT00221897. The patients/participants provided their written informed consent to participate in this study.

## Author Contributions

JR and LZ: conceptualization. JR, LZ, AT, CB, and NE-N: study design. LZ, MF, and RW: funding. JR, LZ, MF, CB, NE-N, AB, MC, JJ, and RW: data interpretation. LZ: supervision. JR, LZ, AT, and MC: data curation. JR, NB, JP, and LZ: visualization. JR, AT, CB, NE-N, MC, and LZ: writing (draft and editing). AT, CB, AB, NB, JP, MF, and NE-N: data analysis. AB, MC, NB, JP, MF, JJ, and RW: writing (editing). JP: methodology. RW: data acquisition. All authors contributed to the article and approved the submitted version.

## Funding

Supported in part by National Eye Institute, EY027510, EY029058, EY026574, K99EY030942, P30EY022589, JR: German Research Foundation research fellowship grant recipient (RE 4155/1-1) and German Ophthalmological Society Grant, University of California, San Diego School of Medicine Summer Research Training Fellowship grant and an unrestricted grant from Research to Prevent Blindness, New York, NY.

## Conflict of Interest

RW Consultant: Abbvie, Aerie Pharmaceuticals, Allergan, Equinox, Eyenovia, Iantrek, Implandata, Ioptic, Nicox, Topcon Financial support: Carl Zeiss Meditec., Bausch & Lomb, Konan, Centervue, Optos, Heidelberg Engineering, National Eye Institute, National Institute for Minority Health and Health Disparities, Optovue, Centervue, Topcon Patent: Toromedes, Meditec-Zeiss. LZ Financial Support: National Eye Institute, Carl Zeiss Meditec Inc., Heidelberg Engineering GmbH, Optovue Inc., Topcon Medical Systems Inc. Patent: Zeiss Meditec. Abbvie (Consultant). The remaining authors declare that the research was conducted in the absence of any commercial or financial relationships that could be construed as a potential conflict of interest.

## Publisher's Note

All claims expressed in this article are solely those of the authors and do not necessarily represent those of their affiliated organizations, or those of the publisher, the editors and the reviewers. Any product that may be evaluated in this article, or claim that may be made by its manufacturer, is not guaranteed or endorsed by the publisher.
